# A Novel QTL and a Candidate Gene Are Associated with the Progressive Motility of Franches-Montagnes Stallion Spermatozoa after Thaw

**DOI:** 10.3390/genes12101501

**Published:** 2021-09-25

**Authors:** Annik Imogen Gmel, Dominik Burger, Markus Neuditschko

**Affiliations:** 1 Animal GenoPhenomics, Agroscope, Route de la Tioleyre 4, 1725 Posieux, Switzerland; markus.neuditschko@agroscope.admin.ch; 2 Equine Department, Vetsuisse Faculty, University of Zurich, Winterthurerstrasse 260, 8057 Zurich, Switzerland; 3 Swiss Institute of Equine Medicine ISME, Agroscope and University of Bern, Les Longs Prés, 1580 Avenches, Switzerland; dominik.burger@vetsuisse.unibe.ch

**Keywords:** fertility, stallion, single nucleotide polymorphism, semen quality, genome-wide heritability

## Abstract

The use of frozen-thawed semen is an important reproduction tool to preserve the biodiversity of small, native horse breeds such as the Franches-Montagnes (FM). However, not all stallions produce cryotolerant semen with a progressive motility after thaw ≥ 35%. To improve our understanding of the genetic background of male fertility traits in both fresh and frozen-thawed semen, we performed genome-wide association studies (GWAS) on gel-free volume, sperm cell concentration, total sperm count, and progressive motility in fresh and frozen-thawed semen from 109 FM stallions using 335,494 genome-wide single nucleotide polymorphisms (SNPs). We identified one significant (*p* < 1.69 × 10^−7^) quantitative trait locus (QTL) on ECA6 within the *SCN8A* gene for progressive motility after thaw, which was previously associated with progressive motility in boars. Homozygous stallions showed a substantial drop in progressive motility after thaw. This QTL could be used to identify cryointolerant stallions, avoiding the costly cryopreservation process. Further studies are needed to confirm whether this QTL is also present in other horse breeds.

## 1. Introduction

In equine breeding, artificial insemination (AI) is a widely used reproduction method, as it has become feasible to extensively use important stallions for selection [1]. Furthermore, AI limits the risks of serious injury to mares and stallions that are often simultaneously valuable, high-performing athletes. Therefore, AI with fresh, cooled-warmed and frozen-thawed semen is routinely applied in European Warmblood breeding, which makes fertility traits related to semen quality economically relevant.

To date, different semen quality traits have been described, including the total number of sperm cells (TSC), the gel-free volume (VOL), the sperm cell concentration in the ejaculate (CON), and the progressive motility of the sperm cells (PM). Except for VOL, these traits have been positively correlated to the pregnancy rate per cycle (PR) in German Warmblood horses [2]. A higher number of healthy sperm cells per ejaculate should improve PR so that mares do not need to be covered again after their initial estrus cycle. Marker-based approaches using microsatellites revealed that variants within the candidate genes *SPATA1, PRLR, ACE, SP17* and *FSHB* were associated with PR in German Warmblood horses [3,4,5]. More recently, additional quantitative trait loci (QTL) have been identified for PR, and for VOL, CON, TSC and PM in German Warmblood horses performing genome-wide association studies (GWAS) on medium-density single nucleotide polymorphism (SNP) arrays (70K SNPs) [6,7].

Compared to European Warmblood breeds, in small, native horse breeds, natural cover is the main reproduction method, to avoid inbreeding by limiting the number of progenies per sire. In the Franches-Montagnes (FM) horse breed native to Switzerland, AI is mostly used with frozen-thawed semen to maintain genetic diversity by preserving endangered sire lines. However, the freezing of semen (cryopreservation) has a negative impact on fertility (reviewed in [8,9]). A common trait to evaluate semen quality in cryopreserved semen is progressive motility after thaw (PMAT), which decreases due to cryopreservation, but should still attain 35% at the minimum to qualify as fertile semen based on current industry recommendations (discussed in [10]). However, despite the ongoing progress in cryopreservation, with widely standardized protocols, in practice not all ejaculates qualify for freezing and thawing, without apparent reason, warranting further research [11,12].

It has already been demonstrated in German Warmblood stallions that PMAT is heritable (*h*^2^ = 0.13 ± 0.04) and genetically correlated to VOL (*r* = −0.30 ± 0.07), CON (*r* = 0.52 ± 0.14), and PM in fresh semen (*r* = 0.39 ± 0.08) [13]. The genetic architecture of PMAT however has not been previously investigated, whilst VOL, CON and PM have only been studied using microsatellites or medium-density SNP data [3,4,5,6,7]. Therefore, the aim of this study was to perform a GWAS of semen quality traits including the PMAT of FM stallions, using high-density SNP data.

## 2. Materials and Methods

### 2.1. Phenotypes

In this study, we retrospectively investigated five semen quality traits of 109 FM stallions including VOL, CON, TSC, PM and PMAT. The data material spanned from the year 1993 to 2021, on semen collected for subsequent cryopreservation from stallions aged 3 to 26 years (median = 5). All stallions were trained to mount a phantom, and semen was collected in an artificial vagina (Avenches model, Switzerland). VOL was measured after the filtration of the ejaculate as gel-free volume. TSC and CON were determined either with the Cell Motion Analyzer (SM-CMA-1074, MTM, Switzerland) [14] or in a Nucleocounter^®^ SP-100 (ChemoMetec, Allerød, Denmark) [15,16]. The gel-free semen was prepared for freezing following the protocol used by Janett et al. [14]. PM (before freezing), and PMAT were determined either with the Cell Motion Analyzer or a computer-assisted sperm analyzer (CASA; HTM-IVOS, Version 12, Beverly, MA, USA).

### 2.2. Raw Data Transformation

In total, 109 stallions were sampled 1–89 times (mean = 12.23) over several weeks, months and years. Due to this sampling bias, and the high variance in previously published datasets (e.g., [17,18,19]), we excluded outliers for each variable within each stallion (exceeding the standard deviation by a factor of 1.5) and calculated the mean for one month within the most recent available year using R v4.1.0 [20]. The month was chosen in order of frequency of available ejaculate data: December–January–February–November–March–April, calculating a monthly average from the earliest available month for each stallion.

### 2.3. Genotypes

The genotypes of the 109 FM stallions were derived from three platforms: 59 stallions were genotyped on the commercial Axiom™ Equine Genotyping Array containing 670,795 evenly distributed markers. For three stallions, whole genome sequence data were available at 10 × coverage, while for the 47 remaining stallions genotyped on the Illumina Equine SNP50 BeadChip^®^ imputed sequence-level genotypes were used, as previously described [21]. The three datasets were merged using PLINK software v1.07 [22] by extracting only shared SNPs, mapped on the new reference genome (EquCab 3.0 [23]) and located on the autosomes (*n* = 479,600 SNPs). Furthermore, we removed SNPs with minor allele frequencies (MAF) below 5%, a SNP genotyping rate below 90%, and those departing from Hardy–Weinberg equilibrium (HWE) at *p* < 0.0001, resulting in 335,494 SNPs for GWAS.

### 2.4. Genetic Analyses

Genome-wide association studies were performed on the continuous variables VOL, CON, TSC, PM and PMAT using a polygenic model approach (*polygenic_hgml*) in the R package GenABEL [24] in R v3.4.1 [20]. The fixed effects age at sampling (AGE), month of sampling (MOS) and year of sampling (YOS) were included in the final models if they reached the significance threshold in the model (*p* < 0.05 using the *summary* function) [25]. The significance of each SNP was extracted using *mmscore*. We visualized the results using Manhattan plots and considered a *p*-value of 10^−5^ for suggestive associations, and determined the significance threshold for the effective number of independent loci (p_ind_) by pruning the 335,494 SNPs for linkage disequilibrium (LD) using a 50 kb sliding window size, a 5 kb window step size and an *r*^2^ exclusion threshold of 0.5. The significance threshold p_ind_ equaled the *p*-value of 0.05 divided by the 109,703 independent SNPs (p_ind_ < 4.56 × 10^−7^) [25,26]. The subset of independent SNPs was also used to estimate the genome-wide heritability of all traits using GCTA software [27]. Finally, we investigated which genes were located near significant SNPs using the NCBI Genome Data Viewer, based on the EquCab 3.0 reference genome assembly [23].

## 3. Results

### 3.1. Phenotypes and Heritabilities

Descriptive statistics of semen quality traits are presented in Table 1 (summary statistics for the overall dataset can be found in Table S1). All traits were highly variable, with high standard deviations (11 to 53% of the mean value). VOL and TSC had the highest standard deviations in percent of the mean value (53 and 48%, respectively), while PM had the lowest standard deviation in percent of the mean value (11%). The sperm quality traits showed low (*h*^2^ < 0.00) to medium (*h*^2^ > 0.40) heritabilities, but with very high standard errors (SE > 0.20). VOL had the highest heritability (*h*^2^ = 0.63 ± 0.26), while PM had the lowest heritability (*h*^2^ = 0.00 ± 0.23).

### 3.2. Genome-Wide Association Study

#### 3.2.1. Covariate Structure

AGE was significant (*p* < 0.05) for VOL and TSC, YOS for VOL, TSC and PM, while MOS was significant for CON and PM. They were therefore included as covariates in the final GWAS. There were no significant covariates for PMAT, and the GWAS was performed without additional covariates.

#### 3.2.2. Overall Results of the GWAS

We identified one significant and three suggestive associations for PMAT, CON, TSC and VOL with visible effect sizes, described hereafter (summary statistics in Table 2). We found no significant or suggestive associations for PM in our dataset.

#### 3.2.3. Significant Association for Progressive Motility after Thaw

**Figure 1:**
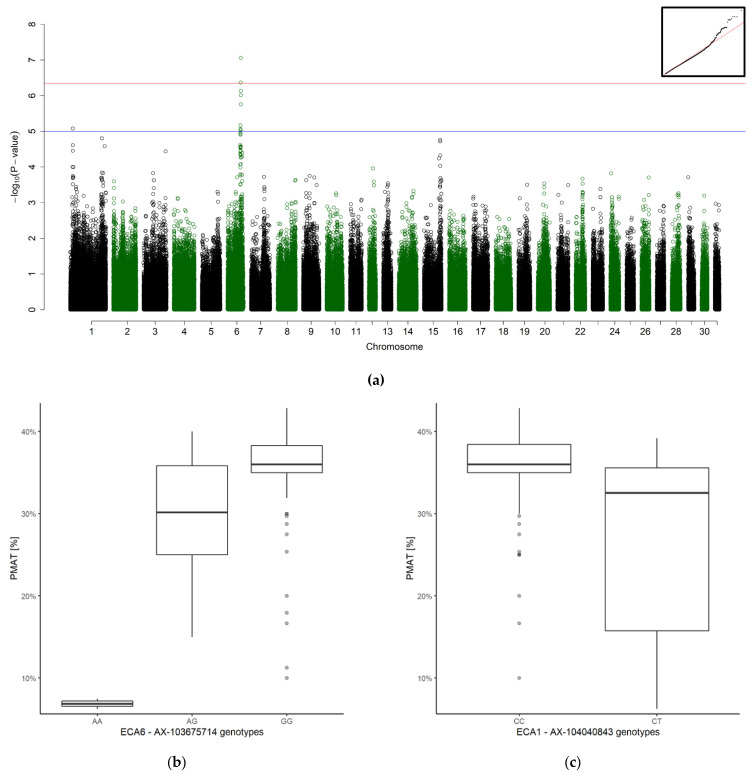
Genome-wide association for progressive motility after thaw (PMAT, *n* = 109). (**a**) Manhattan plot (blue line representing the suggestive significant threshold (*p* < 10^−5^) and (red line) the Bonferroni-corrected significance threshold (p_ind_ < 4.56 × 10^−7^). The inset on the right-hand corner shows the quantile-quantile (Q-Q) plot with the observed *p*-value plotted against the expected one. (**b**,**c**) Boxplots representing the genotype effect of the best associated SNP on ECA6 (**b**) and ECA1 (**c**) on progressive motility after thaw.

The only significant association was with PMAT (Figure 1). The best-associated SNP (ECA6: bp 69,863,974) was located within gene *SCN8A* (sodium voltage-gated channel alpha subunit 8, ECA6: bp 69,728,192–69,904,503). The suggestive SNP on ECA1 (bp 166,470,601) was closest to the gene *NOVA1* (neuro-oncological ventral antigen 1, ECA1: bp 166,624,565–166,773,046).

**Figure 2:**
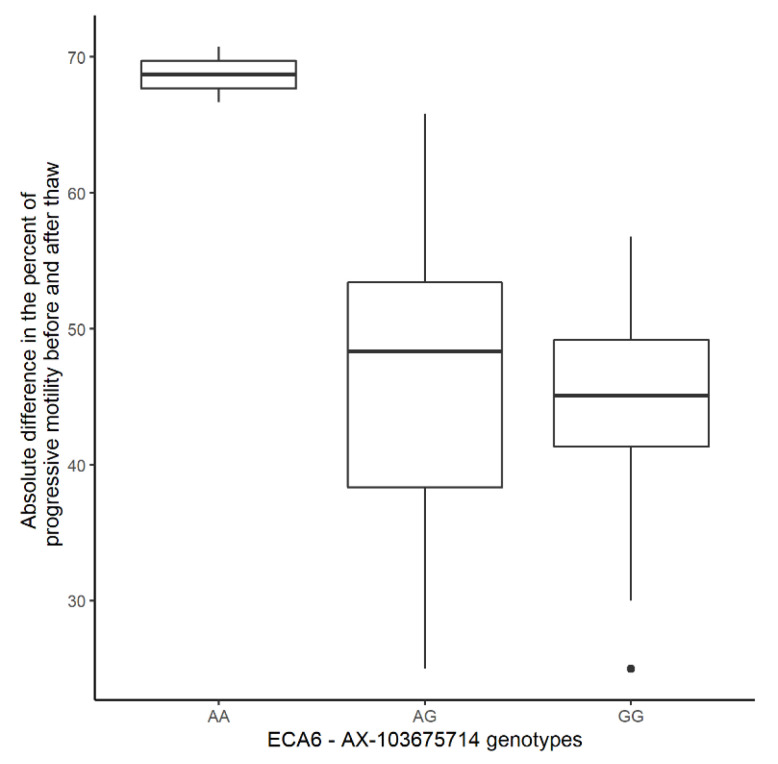
Absolute difference in mean progressive motility between fresh and frozen-thawed semen dependent on the genotype of the best-associated SNP on ECA6, AX-103675714.

Furthermore, we analyzed the difference in PM and PMAT according to the genotype of the best associated SNP (Figure 2). PMAT of the two homozygous affected stallions dropped from a high mean PM of 77.00% and 74.17% to a PMAT of 6.25% and 7.50%, respectively. Low differences between PM and PMAT may indicate either high PM and PMAT, or low PM and PMAT.

#### 3.2.4. Suggestive Results for Other Traits

There was one suggestive association (*p* < 10^−5^, Figure S1) for CON near the gene *ABTB2* (Ankyrin Repeat And BTB Domain Containing 2, ECA12: bp 1,000,419–1,167,693). Another suggestive association was identified for TSC (Figure S2), within the *PTPRT* gene (Protein tyrosine phosphatase receptor type T, ECA22: bp 32,618,040–33,634,569). VOL was suggestively associated with two SNPs (Figure S3), one within the *ZWINT* gene (ZW10 interacting kinetochore protein, ECA1: bp 46,764,308–46,783,553) and one within the *BICRAL* gene (BRD4 interacting chromatin remodeling complex associated protein like, ECA20: bp 42,792,531–42,882,996).

## 4. Discussion

We identified one significant QTL for PMAT and five suggestive QTL for PMAT, CON, TSC and VOL. The best-associated SNP for PMAT was located within the gene *SCN8A*. This particular sodium channel is present in the flagellum and around the neck of mammalian spermatozoa, and thought to be involved in motility [28]. In boars, QTL in the *SCN8A* gene were also associated with motility and progressive motility in fresh semen (frozen semen was not assessed) [29].

The identified QTL for PMAT offers a new perspective on stallions with poor semen freezability, described e.g., in [10,11]. Stallions homozygous for the best-associated SNP within the QTL show a substantial drop in PMAT to a mean of 6.88% despite initially high (>70%) PM, while low PM in fresh semen remains an important indicator for PMAT [10]. Semen doses with mean PM < 70% were less likely to maintain a mean PMAT ≥ 35% after thaw, as PMAT decreases in all stallions due to the freezing process itself [10,11]. Lower initial PM explains five outliers in our GWAS (GG genotypes with low PMAT). Two different GG outliers from the first QTL were heterozygous for the second, albeit only suggestive QTL. Considering the remaining three outliers, additional QTL are likely involved in PMAT, as in the Manhattan plot, at least two additional signals (on ECA1 and ECA15) were apparent, but not reaching the significance threshold. Considering the low prevalence but highly significant effect on semen freezability, young stallions could be genotyped and selected against if they prove to be homozygous, avoiding the costly cryopreservation process. The ejaculates of heterozygous stallions would still need to be evaluated as the mean decrease in PM was less extreme, and individual ejaculates may therefore still qualify for freezing. Furthermore, selecting against heterozygous stallions may have an excessively detrimental effect on conservation programs regarding genetic diversity. Finally, the absence of the A allele alone does not guarantee that PMAT will be high enough for industry standards, considering that other factors such as seasonality and initial PM also play an important role in PMAT [10,14].

The second, suggestive QTL for PMAT on ECA1 was located near the *NOVA1* gene, for which we could find no association to male fertility in the literature. The suggestive SNP on ECA12 for sperm concentration was nearest *ABTB2* gene. Its function in relation to fertility is not well known. However, a recent study suggested that the *ABTB2* gene expression is generally upregulated in normal Sertoli cells compared to those of mice with knocked-out Sertoli cell reprogramming genes *SOX9, DMRT1* or *GATA4* [30]. Sertoli cells produce seminal fluid, which could explain the suggestive association between the *ABTB2* gene and sperm concentration in our equine study. The second suggestive association for concentration on ECA1 was near the *ADGRA1* gene. The G protein-coupled receptor superfamily contains at least two genes, *ADGRA2* and *ADGRG1*, involved in male fertility [31]. *ADGRG1* was expressed in the Sertoli cells, with testis development defective in knockout mice [32]. *ADGRA2* knockout mice showed sperm with very low motility [33]. The function of *ADGRA1* however remains unclear. Although both QTL for sperm concentration were near genes essential for Sertoli cell functioning, these SNPs were not associated with VOL or TSC. This is likely due to the suggestive and not significant statistical associations, the large variance and the relatively small sample in this study. *BRD4,* the gene to which *BICRAL* is associated, is involved in spermatogenesis [34], but so far has not been associated with seminal fluid, thus our association of the QTL within *BICRAL* with VOL remains speculative. The *ZWINT* (or *ZW10*) gene has essential functions in the meiotic process [35], but other effects on male fertility have not been reported in horses. The *PTPRT* protein was expressed in the equine spermatozoa proteome [36], but its exact function is also unknown.

Compared to the most recent study on Warmblood stallions, mean VOL and TSC were lower (25 vs. 37 ml, respectively 6 vs. 7 × 10^9^), and CON, PM and PMAT higher (286 vs. 213 × 10^6^/mL; 79 vs. 61%; 35 vs. 33%) [13]. This may be caused by breed-specific or methodological differences in semen collection. Interestingly, in the mean, PMAT was slightly below the suggested threshold for a PMAT of 35% in German Warmblood stallions, while mean PM was approximately 15% higher in FM compared to Warmblood stallions [13]. We could not confirm any previously reported QTL in Warmblood horses, which suggests that different QTL are involved in the fertility of FM and Warmblood horses. This warrants further investigations into PMAT in Warmblood stallions, where AI use is more common.

Despite the relatively small sample size of only 109 stallions, this sample still represents nearly half of all FM stallions available for reproduction (235 active stallions in 2021, including frozen sperm from deceased stallions). In contrast to previous studies [14,16,19], MOS (i.e., seasonality) did not significantly affect VOL, TSC or PMAT, which may at least partially be due to the fact that we did not use any samples from the summer months (June to August). AGE had an effect on TSC, but not on PMAT, contrary to [10] but similar to [19]. Changes in data collection methods are confounded within the covariate YOS, which affected VOL, TSC and PM. The absence of significant or suggestive QTL for PM could be at least partially due to higher uncertainty in the phenotype. At the reproduction center, semen evaluations of FM stallions are essentially performed to ensure that cryopreservation is possible, therefore, more care is placed on accurately quantifying PMAT than PM. Other reported factors with an influence on fertility, such as the use of deworming medication [37] or the histocompatibility of teaser mares [15] were not systematically recorded during semen collection and could therefore not be corrected for. However, considering that PMAT was not significantly affected by any of the covariates of AGE, MOS, or YOS, this further validates our results for this trait.

## 5. Conclusions

We identified a novel QTL in horses that affects PMAT but not PM. This information could be used to pre-screen stallions destined for AI and cryopreservation in particular. However, this QTL needs to be confirmed in other breeds before the widespread use of marker-assisted selection.

## Figures and Tables

**Table 1:** Mean, standard deviation (SD), minimum, maximum, genome-wide heritability and standard error for 109 stallions of the semen quality traits.

Trait	Mean	SD	Min	Max	*h* ^2^	SE
VOL	24.48	12.91	4.00	70.50	0.63	0.26
CON	284.00	91.99	122.80	580.00	0.48	0.27
TSC	6.12	2.94	1.81	17.34	0.02	0.24
PM	80.11	8.51	50.00	90.00	0.00	0.23
PMAT	34.51	7.32	6.25	45.33	0.03	0.21

VOL: gel-free volume (ml), CON: sperm concentration in the gel-free volume (10^6^ spermatozoa/ml), TSC: total sperm count (10^9^ spermatozoa) = gel-free volume × sperm concentration, PM: progressive motility [%], PMAT: progressive motility after thaw (%).

**Table 2:** Best association by trait (only markers with *p* < 10^−5^ are reported) for the genome-wide association studies of sperm quality traits in 109 Franches-Montagnes stallions.

Trait	ECA	Position EquCab 3.0	#SNP	SNP with the Lowest *p*-Value	*p*-Value	Number of Genotypes	Genotypes of Stallions			Reference Allele	Alternate Allele
							HOM_R_	HET	HOM_A_		
PMAT	6	67,290,346–69,885,065	11	AX-103675714	1.69 × 10^−7^	108	95	11	2	G	A
PMAT	1	166,470,601	1	AX-104040843	7.45 × 10^−6^	109	95	14	0	C	T
CON	12	1,050,320	1	AX-103675085	2.09 × 10^−6^	105	92	12	1	C	T
TSC	22	33,024,116	1	AX-103616662	6.71 × 10^−6^	109	31	52	26	C	T
VOL	1	46,782,612	1	AX-104100234	8.86 × 10^−6^	109	91	18	0	G	A
VOL	20	42,868,232	1	AX-104083513	9.82 × 10^−6^	108	1	13	94	A	G

#SNP: the number of single nucleotide polymorphisms for a specific trait that passed the suggestive p-value threshold of 10^−5^, P-value: p-value of the SNP with the lowest p-value per trait and locus corrected for genomic inflation (Pc1df from GenABEL), HOM_R_: homozygous for the reference allele, HET: heterozygous, HOM_A_: homozygous for the alternate allele, PMAT: progressive motility after thaw, CON: concentration, TSC: total sperm count, VOL: gel-free volum.

## Data Availability

The phenotypic data in this study concern both stallions owned by the Swiss national stud farm and private clients to the reproduction center, and are therefore not publicly available. Genotypic data are available under reasonable request addressed to the authors.

## Supplementary Materials

The following are available online at https://www.mdpi.com/article/10.3390/genes12101501/s1, Table S1: Mean, standard deviation (SD), minimum, maximum, and standard error for 109 stallions of the recorded semen quality traits for 1039 ejaculates, Figure S1: Genome-wide association for concentration, Figure S2: Genome-wide association for total sperm count, Figure S3: Genome-wide association for gel-free volume.

## Animal Testing Permit

The study was conducted according to Swiss legislation under the permit VD2227.2.

## References

[1] Miller C. (2008). Optimizing the use of frozen–thawed equine semen. Theriogenology.

[2] Gottschalk M., Sieme H., Martinsson G., Distl O. (2017). Relationships among stallion fertility and semen traits using estimated breeding values of German Warmblood stallions. Theriogenology.

[3] Giesecke K., Hamann H., Stock K., Woehlke A., Sieme H., Distl O. (2009). Evaluation of SPATA1-associated markers for stallion fertility. Anim. Genet..

[4] Giesecke K., Hamann H., Stock K., Klewitz J., Martinsson G., Distl O., Sieme H. (2011). Evaluation of ACE, SP17, and FSHB as candidates for stallion fertility in Hanoverian warmblood horses. Anim. Reprod. Sci..

[5] Giesecke K., Hamann H., Sieme H., Distl O. (2010). Evaluation of prolactin receptor (PRLR) as candidate gene for male fertility in Hanoverian warmblood horses. Reprod. Domest. Anim..

[6] Gottschalk M., Metzger J., Martinsson G., Sieme H., Distl O. (2016). Genome-wide association study for semen quality traits in German Warmblood stallions. Anim. Reprod. Sci..

[7] Schrimpf R., Dierks C., Martinsson G., Sieme H., Distl O. (2014). Genome-wide association study identifies phospholipase C zeta 1 (PLCz1) as a stallion fertility locus in Hanoverian warmblood horses. PLoS ONE.

[8] Watson P.F. (2000). The causes of reduced fertility with cryopreserved semen. An. Reprod. Sci..

[9] Holt W. (2000). Basic aspects of frozen storage of semen. Anim. Reprod. Sci..

[10] Aurich J., Kuhl J., Tichy A., Aurich C. (2020). Efficiency of semen cryopreservation in stallions. Animals.

[11] Yeste M., Estrada E., Rocha L.G., Marín H., Rodríguez-Gil J.E., Miró J. (2015). Cryotolerance of stallion spermatozoa is related to ROS production and mitochondrial membrane potential rather than to the integrity of sperm nucleus. Andrology.

[12] Graham J.K. (1996). Cryopreservation of stallion spermatozoa. Vet. Clin. N. Am. Equine Pract..

[13] Greiser T., Sieme H., Martinsson G., Distl O. (2019). Genetic parameters and estimated breeding values for traits of raw and frozen-thawed semen in German Warmblood stallions. Anim. Reprod. Sci..

[14] Janett F., Thun R., Bettschen S., Burger D., Hassig M. (2003). Seasonal changes of semen quality and freezability in Franches–Montagnes stallions. Anim. Reprod. Sci..

[15] Jeannerat E., Marti E., Berney C., Janett F., Bollwein H., Sieme H., Burger D., Wedekind C. (2018). Stallion semen quality depends on major histocompatibility complex matching to teaser mare. Mol. Ecol..

[16] Wach-Gygax L., Burger D., Malama E., Bollwein H., Fleisch A., Jeannerat E., Thomas S., Schuler G., Janett F. (2017). Seasonal changes of DNA fragmentation and quality of raw and cold-stored stallion spermatozoa. Theriogenology.

[17] Gottschalk M., Sieme H., Martinsson G., Distl O. (2016). Analysis of breed effects on semen traits in light horse, warmblood, and draught horse breeds. Theriogenology.

[18] Labitzke D., Sieme H., Martinsson G., Distl O. (2014). Genetic Parameters and Breeding Values for Semen Characteristics in H anoverian Stallions. Reprod. Domest. Anim..

[19] Greiser T., Sieme H., Martinsson G., Distl O. (2020). Breed and stallion effects on frozen-thawed semen in warmblood, light and quarter horses. Theriogenology.

[20] R Core Team (2013). R: A Language and Environment for Statistical Computing.

[21] Frischknecht M., Neuditschko M., Jagannathan V., Drögemüller C., Tetens J., Thaller G., Leeb T., Rieder S. (2014). Imputation of sequence level genotypes in the Franches-Montagnes horse breed. Genet. Sel. Evol..

[22] Purcell S., Neale B., Todd-Brown K., Thomas L., Ferreira M.A., Bender D., Maller J., Sklar P., De Bakker P.I., Daly M.J. (2007). PLINK: A tool set for whole-genome association and population-based linkage analyses. Am. J. Hum. Genet..

[23] Kalbfleisch T.S., Rice E., DePriest M.S., Walenz B.P., Hestand M.S., Vermeesch J.R., O’Connell B.L., Fiddes I.T., Vershinina A.O., Petersen J.L. (2018). EquCab3, an Updated Reference Genome for the Domestic Horse. bioRxiv.

[24] Aulchenko Y.S., Ripke S., Isaacs A., Van Duijn C.M. (2007). GenABEL: An R library for genome-wide association analysis. Bioinformatics.

[25] Gmel A.I., Druml T., von Niederhäusern R., Leeb T., Neuditschko M. (2019). Genome-wide association studies based on equine joint angle measurements reveal new QTL affecting the conformation of horses. Genes.

[26] Kanai M., Tanaka T., Okada Y. (2016). Empirical estimation of genome-wide significance thresholds based on the 1000 Genomes Project data set. J. Hum. Genet..

[27] Yang J., Lee S.H., Goddard M.E., Visscher P.M. (2011). GCTA: A tool for genome-wide complex trait analysis. Am. J. Hum. Genet..

[28] Pinto F.M., Ravina C.G., Fernández-Sánchez M., Gallardo-Castro M., Cejudo-Román A., Candenas L. (2009). Molecular and functional characterization of voltage-gated sodium channels in human sperm. Reprod. Biol. Endocrinol..

[29] Marques D.B., Bastiaansen J.W., Broekhuijse M.L., Lopes M.S., Knol E.F., Harlizius B., Guimarães S.E., Silva F.F., Lopes P.S. (2018). Weighted single-step GWAS and gene network analysis reveal new candidate genes for semen traits in pigs. Genet. Sel. Evol..

[30] Rahmoun M., Lavery R., Laurent-Chaballier S., Bellora N., Philip G.K., Rossitto M., Symon A., Pailhoux E., Cammas F., Chung J. (2017). In mammalian foetal testes, SOX9 regulates expression of its target genes by binding to genomic regions with conserved signatures. Nucleic Acids Res..

[31] Langenhan T. (2020). Adhesion G protein–coupled receptors—Candidate metabotropic mechanosensors and novel drug targets. Basic Clin. Pharmacolo. Toxicol..

[32] Chen G., Yang L., Begum S., Xu L. (2010). GPR56 is essential for testis development and male fertility in mice. Dev. Dyn..

[33] Davies B., Baumann C., Kirchhoff C., Ivell R., Nubbemeyer R., Habenicht U.-F., Theuring F., Gottwald U. (2004). Targeted deletion of the epididymal receptor HE6 results in fluid dysregulation and male infertility. Mol. Cell. Biol..

[34] Bryant J.M., Donahue G., Wang X., Meyer-Ficca M., Luense L.J., Weller A.H., Bartolomei M.S., Blobel G.A., Meyer R.G., Garcia B.A. (2015). Characterization of BRD4 during mammalian postmeiotic sperm development. Mol. Cell. Biol..

[35] Williams B.C., Gatti M., Goldberg M.L. (1996). Bipolar spindle attachments affect redistributions of ZW10, a Drosophila centromere/kinetochore component required for accurate chromosome segregation. J. Cell Biol..

[36] Swegen A., Aitken R. (2014). Characterisation of the stallion sperm proteome. J. Equine Vet. Sci..

[37] Janett F., Thun R., Ryhiner A., Burger D., Hassig M., Hertzberg H. (2001). Influence of Eqvalan^®^(ivermectin) on quality and freezability of stallion semen. Theriogenology.

