# Supplementary material for: A Novel QTL and a Candidate Gene Are Associated with the Progressive Motility of Franches-Montagnes Stallion Spermatozoa after Thaw
Source: Genes (Basel). 2021 Sep 25;12(10):1501. doi: 10.3390/genes12101501 (PMC8536120; doi:10.3390/genes12101501)
Supplement: Supplementary file 1 [file genes-12-01501-s001.zip › SFiles/Figure S2.pdf]

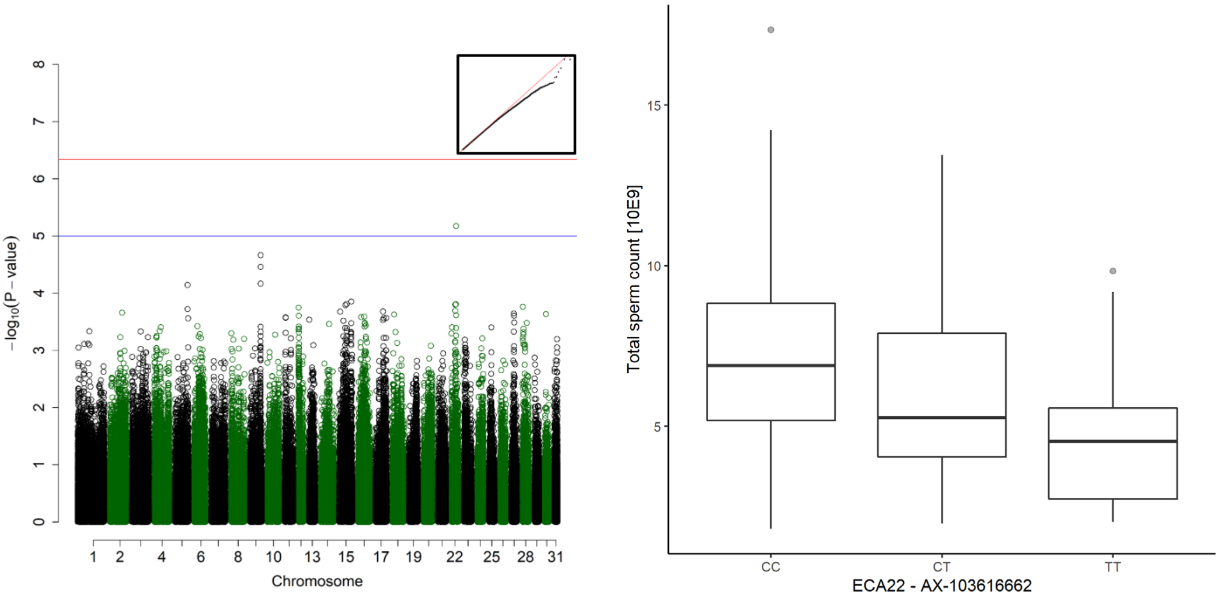

(**a**) (**b**)

Figure S2. *Genome-wide association for total sperm count (n=109).* ***(a)*** *Manhattan plot (blue line representing the suggestive significance threshold ($p<10^{-5}$) and (red line) the Bonferroni-corrected significance threshold ($p<4.56 \times 10^{-7}$). The inset on the right hand corner shows the quantile-quantile (Q-Q) plot with the observed p-value plotted against the expected one.* ***(b)*** *Boxplot representing the genotype effect of the best associated SNP on ECA22.*
