# Supplementary material for: A Novel QTL and a Candidate Gene Are Associated with the Progressive Motility of Franches-Montagnes Stallion Spermatozoa after Thaw
Source: Genes (Basel). 2021 Sep 25;12(10):1501. doi: 10.3390/genes12101501 (PMC8536120; doi:10.3390/genes12101501)
Supplement: Supplementary file 1 [file genes-12-01501-s001.zip › SFiles/Figure S3.pdf]

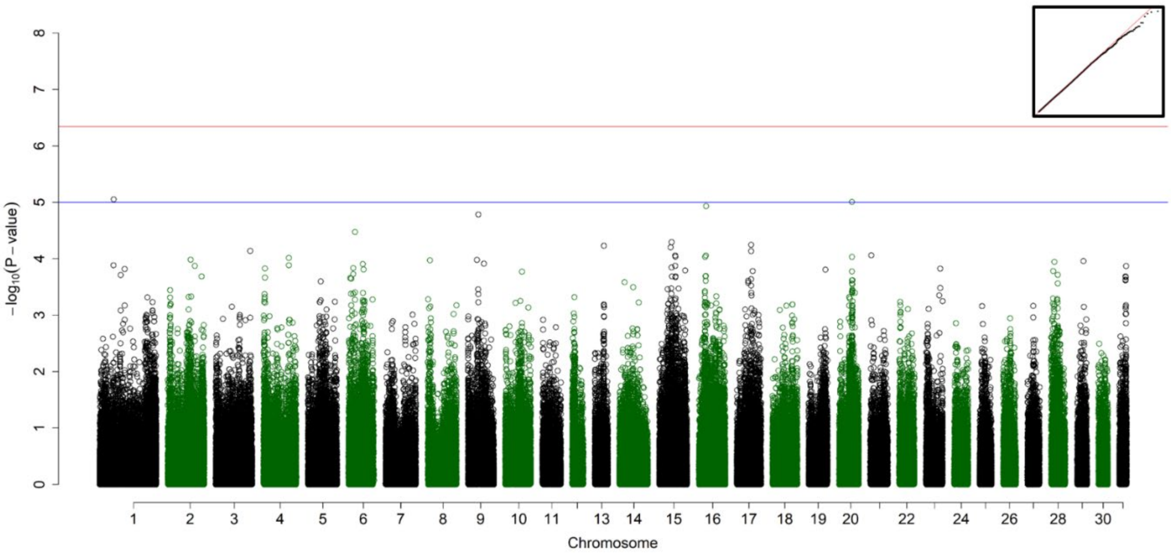

(a)

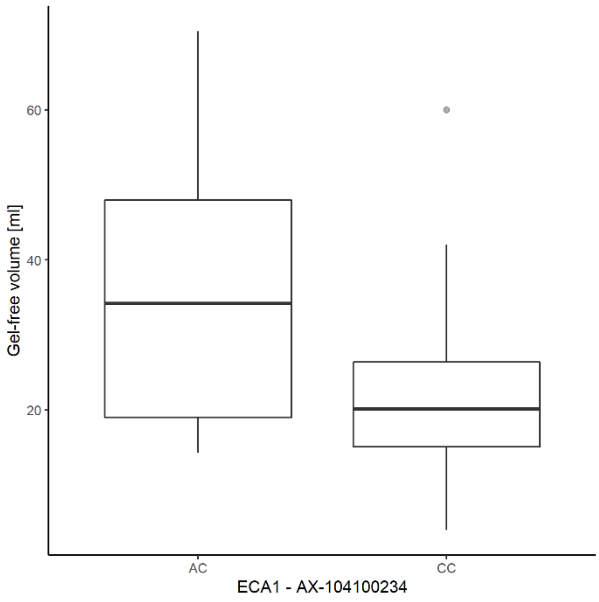

(**b**)

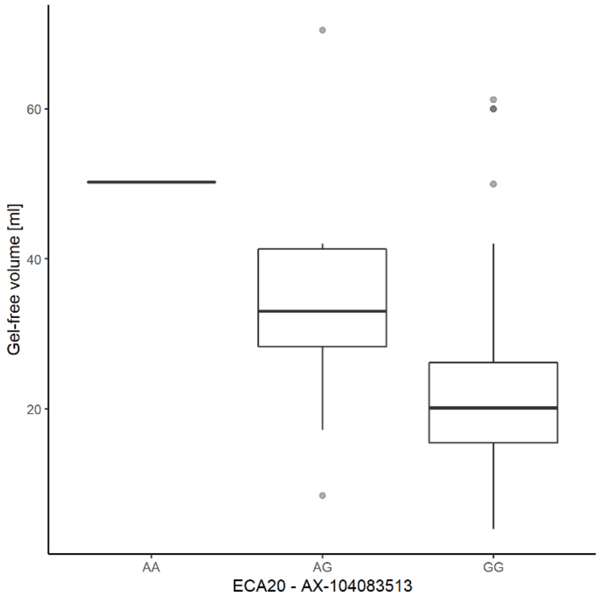

(**c**)

Figure S3. *Genome-wide association for gel-free volume (VOL, n=109).* ***(a)*** *Manhattan plot (blue line representing the suggestive significance threshold ($p<10^{-5}$) and (red line) the Bonferroni-corrected significance threshold ($p<4.56 \times 10^{-7}$). The inset on the right hand corner shows the quantile-quantile (Q-Q) plot with the observed p-value plotted against the expected one.* ***(b,c)*** *boxplots representing the genotype effect of the best associated SNP on ECA1 (b) and ECA20 (c) on the gel-free volume.*
