# Supplementary material for: A Novel QTL and a Candidate Gene Are Associated with the Progressive Motility of Franches-Montagnes Stallion Spermatozoa after Thaw
Source: Genes (Basel). 2021 Sep 25;12(10):1501. doi: 10.3390/genes12101501 (PMC8536120; doi:10.3390/genes12101501)
Supplement: Supplementary file 1 [file genes-12-01501-s001.zip › SFiles/Table S1.pdf]

Table S1. Mean, standard deviation (SD), minimum, maximum, and standard error for 109 stallions of the recorded semen quality traits for 1039 ejaculates

**Trait**	**Mean**	**SD**	**Min**	**Max**
VOL [ml]	22.35	13.53	2.00	100.00
CON [10^6/ml]	282.60	103.98	42.00	615.00
TSC [10^9]	5.75	3.11	0.58	23.93
PM [%]	80.07	8.76	50.00	90.00
PMAT [%]	34.84	8.45	5.00	60.00

VOL: gel-free volume [ml], CON: Sperm concentration in the gel-free volume [10^6/ml], TSC: Total sperm count [10^9], PM: Progressive motility [%], PMAT: Progressive motility after thaw [%]
